# Healthcare costs of cutaneous melanoma according to comorbidity patterns: a population-based study from the Regional Cancer Registry of the Veneto Region

**DOI:** 10.3389/fpubh.2025.1668198

**Published:** 2025-10-17

**Authors:** Alessandra Buja, Fortunato Cassalia, Massimo Rugge, Chiara Trevisiol, Manuel Zorzi, Paolo Del Fiore, Ilaria Pantaleo, Carlo Riccardo Rossi, Pierfranco Conte, Anna Belloni Fortina, Simone Mocellin

**Affiliations:** ^1^Department of Cardiac, Thoracic, Vascular Sciences, and Public Health, University of Padova, Padova, Italy; ^2^Department of Medicine - DIMED, Dermatology Unit, University of Padova, Padova, Italy; ^3^Department of Medicine – DIMED, Pathology and Cytopathology Unit, University of Padova, Padova, Italy; ^4^Soft-Tissue, Peritoneum, and Melanoma Surgical Oncology Unit, Veneto Institute of Oncology IOV-IRCCS, Padova, Italy; ^5^Veneto Tumour Registry (RTV), Azienda Zero, Padova, Italy; ^6^Department of Surgery, Oncology, and Gastroenterology – DISCOG, University of Padova, Padova, Italy; ^7^IRCCS San Camillo Hospital, Venezia, Italy; ^8^Periplo Foundation, Cremona, Italy; ^9^Department of Women’s and Children’s Health (SDB), Pediatric Dermatology Regional Center, University of Padova, Padova, Italy

**Keywords:** melanoma, comorbidity, costs, health care resources, health care services, cancer registries, cohort study

## Abstract

**Background:**

Cutaneous malignant melanoma represents a notable public health issue, characterized by a rapidly increasing incidence, particularly among younger populations. Despite progress in early detection and treatment modalities, this rising trend exacerbates the healthcare system’s burden. Limited research has been conducted on the impact of comorbidities on overall and melanoma-specific healthcare costs incurred by patients with melanoma. The objective of this study is to assess how various comorbidity patterns influence healthcare costs in this patient population.

**Methods:**

This retrospective cohort study reviewed data from the Regional Cancer Registry of the Veneto Region (RTV) for melanoma diagnoses in 2019 and 2021. Patients were grouped into specific comorbidity clusters using latent class analysis, and the effect of these patterns on melanoma healthcare costs was evaluated from a health system perspective, considering only the direct costs incurred by the regional health care service.

**Results:**

The study included 2,978 cases of incident melanoma. The 2,114 patients with comorbidity data available were categorized into three comorbidity clusters: Circulatory-Metabolic-Respiratory, Psychosocial-Pregnancy related, and Multiorgan-Trauma. The mean unadjusted overall and melanoma-specific cumulative expenditure per patient increased with the number of comorbidities: melanoma-specific healthcare resources were € 13,537 (no comorbidity), € 16,828 (one comorbidity), € 20,396 (Multiorgan-Trauma cluster). Hospitalizations were the primary driver of cost escalation, particularly for patients with Multiorgan-Trauma comorbidities.

**Conclusion:**

Comorbidity patterns significantly impact melanoma management and related healthcare costs. Understanding these patterns can help optimize resource allocation and improve patient management strategies.

## 1 Introduction

Cutaneous malignant melanoma is a significant public health problem with a rapidly increasing incidence worldwide, particularly in younger populations (1, 2). The increasing incidence of melanoma poses a significant and multifaceted burden on healthcare systems worldwide. Despite early detection, the growing number of cases and new therapies strain medical resources and finances (3, 4). The economic burden of melanoma could be significantly amplified when patients present with comorbidities, which can increase treatment costs and complicate care management, creating a complex landscape for healthcare systems (5). A study found that melanoma patients with comorbidities incurred significantly higher healthcare costs compared to those without (6). Another study found that cardiovascular diseases, in particular, have been associated with increased healthcare utilization and costs among non-melanoma patients (7). In fact, the presence of comorbidities often necessitates additional medications, more frequent medical visits, and longer hospital stays, all contributing to elevated expenses. Moreover, comorbidities can impact treatment decisions and efficacy, potentially leading to more costly interventions or extended treatment durations (8, 9).

The problem of melanoma is exacerbated when it affects patients with multiple comorbidities. These conditions can significantly complicate the management of melanoma, leading to delayed diagnosis, altered treatment decisions, and worse clinical outcomes. In addition, comorbidities often require additional medical interventions, resulting in higher overall medical costs, and the interplay between the complexity of melanoma treatment and the management of comorbidities therefore presents unique challenges (10–12). Patients with multiple chronic conditions tend to have more advanced stages of melanoma, in part due to the complexity associated with managing numerous conditions simultaneously (9, 13, 14). Despite the significant impact of comorbidities on clinical outcomes and healthcare costs, the current literature lacks a comprehensive economic evaluation of melanoma care that considers specific comorbidity profiles.

This study aims to address this gap by utilizing data from the Veneto Region of Italy to provide a detailed assessment of melanoma-related healthcare costs, taking into account the impact of various comorbidity patterns.

## 2 Methods

### 2.1 Study population

This population-based retrospective cohort study included 2,114 melanoma patients with a confirmed diagnosis of melanoma as recorded in the high-resolution melanoma Veneto Regional Cancer Registry (RTV) (15) in 2019 and 2021 and with available comorbidity data. The RTV is a certified, population-based cancer registry recording all malignancies diagnosed in the region’s residents, who number approximately 4.9 million (16). The recording procedures rely on an integrated information network that includes pathology reports (including pT-, pN-, and M values and the resulting pTNM-AJCC stage; 8th edition) (17), clinical charts, death certificates, and public health administrative records. Mortality data of the patients were tracked by linking RTV’s digital records with those from the regional mortality registration, which captures events occurring outside the regional territory (18).

### 2.2 Comorbidities

Information regarding comorbidities was sourced from hospital records, which included primary and secondary diagnoses, recorded either prior to or within 6 months following the diagnosis. Patients lacking hospital records were excluded from the study. Seventeen primary major disease categories were analyzed (major disease categories and V codes). The melanoma patients were categorized into five groups based on comorbidity burden: no comorbidities (Comorbidity 0), presence of one comorbidity (Comorbidity 1), and those with two or more concurrent diseases, other than melanoma, which were categorized according to three different comorbidity patterns. These patterns were identified using Latent Class Analysis (LCA), with the optimal number of classes being determined by the Akaike Information Criterion (AIC) (19). The model fit was optimal at three classes (Classes 1, 2 and 3; lowest AIC, entropy = 0.82). For clinical interpretability, the three multimorbidity clusters derived from latent class analysis were labelled according to their dominant major comorbidity categories: Class 1 as “Circulatory-Metabolic-Respiratory,” Class 2 as “Psychosocial-Pregnancy related” and Class 3 as “Multiorgan-Trauma” diseases (Table 1).

**Table 1:** Probability (%) that a patient belongs to latent classes 1, 2, and 3 for each disease group.

Disease	Class 1	Class 2	Class 3
	Circulatory-metabolic-respiratory	Psychosocial-pregnancy related	Multiorgan-trauma
Diseases of the circulatory system	**96.44%**	2.75%	50.29%
Endocrine, nutritional, metabolic diseases	**53.20%**	4.93%	28.61%
Diseases of the respiratory system	**32.15%**	1.38%	25.21%
Factors influencing health status (V codes)	60.71%	**98.59%**	39.53%
Complications of pregnancy, childbirth	0.00%	**23.84%**	0.00%
Diseases of the skin and subcutaneous tissue	0.00%	**15.86%**	8.75%
Trauma and poisoning	2.25%	15.65%	**31.70%**
Diseases of the genitourinary system	19.31%	13.14%	**30.30%**
Diseases of the digestive system	5.51%	7.31%	**25.69%**
Diseases of the blood and hematopoietic organs	0.00%	2.43%	**23.75%**
Symptoms, signs, ill-defined conditions	9.17%	11.67%	**20.98%**
Diseases of the nervous system	3.95%	4.08%	**19.59%**
Diseases of the musculoskeletal system	0.00%	14.27%	**18.45%**
Infectious and parasitic diseases	0.00%	1.49%	**16.56%**
Mental disorders	0.00%	1.89%	**8.55%**
Congenital malformations	0.00%	0.00%	**3.40%**

Bold the highest probability that a patient belongs to one of the three LCA classes for the disease group.

### 2.3 Costs analysis

The cost analysis was evaluated from a health system perspective, considering only the direct costs incurred by the regional health care service and conducted using anonymized aggregate data. For both patient cohorts, the cost estimates encompass a 3-year period following the initial cancer diagnosis. These estimates account for disease-related expenses as provided by the Regional Health Authority. Box 1 outlines the sources and profiles of the administrative data. Each patient was assigned a unique and anonymous identification code, which was used to link all administrative data covering hospital admissions, drug prescriptions, outpatient visits, emergency room visits, hospice admissions, medical devices, and vital statuses. The average per-patient costs were calculated and stratified according to the stage of disease at the time of diagnosis. Melanoma-specific cost items were evaluated in accordance with the healthcare resources compatible with the melanoma-specific clinical pathway (20).

Tobit regression models with hospital-level clustering, left-censored at zero, were used to examine the impact of different comorbidity patterns on both all-cause and melanoma-specific healthcare costs, while adjusting for sex, age, and stage at diagnosis. Cluster-robust standard errors were calculated to account for intra-hospital correlation among patients treated within the same healthcare facility.

The statistical packages R 3.6.2 and SAS 9.4 were used for the record linkage and all statistical analyses.

**BOX 1** Healthcare costs of melanoma patients; administrative regional databases included in the cost estimates.

**Table:** 

Administrative databases	Data collection
Hospital admissions	Defines the Diagnosis-Related Groups (DRGs) for each admission, valued according to an inpatient formulary (i.e., Tariffario Prestazioni Ospedaliere), encompassing all in-hospital activities, including drugs.
Pharmaceutical distribution and hospital drug consumption	Consider the costs of medical therapies (costs calculated on the prescribed doses).
Outpatient visits	Procedures/services provided under regional health service funding at outpatient facilities. Economic values based on rates established by an outpatient formulary (i.e., Tariffario Prestazioni Ambulatoriali).
Emergency room admissions	Costs are based on the rates for all medical procedures and interventions performed during A&E visits.
Hospice admission	Costs are calculated by multiplying a regional daily rate by the number of days spent in hospice.
Medical devices	Reports the expenditures incurred by regional authorities for the provision of medical devices.

### 2.4 Ethics

The study was conducted in accordance with the principles outlined in the Declaration of Helsinki. All data were anonymized following Italian regulations and handled for monitoring and quality assurance purposes. The data analyses were performed on anonymous, aggregated data, ensuring that no individual could be identified. Data processing was conducted in accordance with GDPR-compliant procedures. Ethical approval for the study was obtained from the Veneto Oncological Institute’s ethics committee (no. 52/2016).

## 3 Results

We analyzed 2,114 incident cutaneous melanoma cases diagnosed in 2019 and 2021, 50.4% (*n* = 1,066) had no record of chronic disease, 29.7% (*n* = 628) had one, and 19.9% (*n* = 420) had more than one comorbidity. Latent-class analysis (LCA) Class 1 (Circulatory-Metabolic-Respiratory) included 97 (4.6%) patients with circulatory, endocrine and respiratory diseases as dominant conditions; Class 2 (Psychosocial-Pregnancy related) encompassed 136 (6.4%) patients with factors influencing health status and pregnancy related diseases; Class 3 (Multiorgan-Trauma) comprised 187 (8.8%) patients with multiorgan and trauma related diseases.

Table 2 details the characteristics of sample by comorbidity groups. There are disparities in sex distribution, with males making up the majority of cases, but with varying proportions: 54.1% *versus* 45.9% for those without comorbidities, 62.4% versus 37.6% with one comorbidity, and 80.4% *versus* 19.6% in Class 1. There are also differences in average age, with Classes 1 and 3 having higher average ages. Significant variations in cancer stage at diagnosis were found, with Stage I being more common in patients without comorbidities. Patients with comorbidities were less likely to use medical therapy (*p* = 0.39) especially in Class 1, compared to those without comorbidities. Additionally, only 34% of patients without comorbidities chose not to receive either immunotherapy or targeted therapy compared to 41.5% in Class 3 and 76.9% in Class 1.

**Table 2:** Study population characteristics by comorbidity group.

Variable	No comorbidity	1 comorbidity	Class 1	Class 2	Class 3	*p*-value
			Circulatory-metabolic-respiratory	Psychosocial-pregnancy related	Multiorgan-trauma	
*N*	1,066 (50.4)	628 (29.7)	97 (4.6)	136 (6.4)	187 (8.8)	
Sex (%)						
Male	577 (54.1)	392 (62.4)	78 (80.4)	64 (47.1)	117 (62.6)	<0.001^***^
Female	489 (45.9)	236 (37.6)	19 (19.6)	72 (52.9)	70 (37.4)	
Age (mean (SD))	61.05 (14.92)	63.00 (14.99)	72.70 (11.54)	57.41 (16.65)	73.22 (13.60)	<0.001^***^
Stage (%)						
I	719 (67.4)	374 (59.6)	49 (50.5)	82 (60.3)	98 (52.4)	<0.001^***^
II	178 (16.7)	110 (17.5)	16 (16.5)	28 (20.6)	31 (16.6)	
III	109 (10.2)	93 (14.8)	11 (11.3)	19 (14.0)	14 (7.5)	
IV	29 (2.7)	31 (4.9)	15 (15.5)	6 (4.4)	27 (14.4)	
Missing	31 (2.9)	20 (3.2)	6 (6.2)	1 (0.7)	17 (9.1)	
N (Stage III–IV)	138	124	26	25	41	
Medical therapy *n* (%)						
None	47 (34.1)	54 (43.5)	20 (76.9)	10 (40.0)	17 (41.5)	0.039^*^
Only Immunotherapy	46 (33.3)	30 (24.2)	2 (7.7)	6 (24.0)	12 (29.3)	
Only Target therapy	42 (30.4)	35 (28.2)	4 (15.4)	8 (32.0)	9 (22.0)	
Target therapy & immunotherapy	3 (2.2)	5 (4.0)	0 (0.0)	1 (4.0)	3 (7.3)	
N (Stage III)	109	93	11	19	14	
Lymphadenectomy *n* (%)						
No	98 (89.9)	84 (90.3)	10 (90.9)	19 (100.0)	12 (85.7)	0.654
Yes	11 (10.1)	9 (9.7)	1 (9.1)	0 (0.0)	2 (14.3)	

*p < 0.05; **p < 0.01; ***p < 0.001.

**Figure 1:**
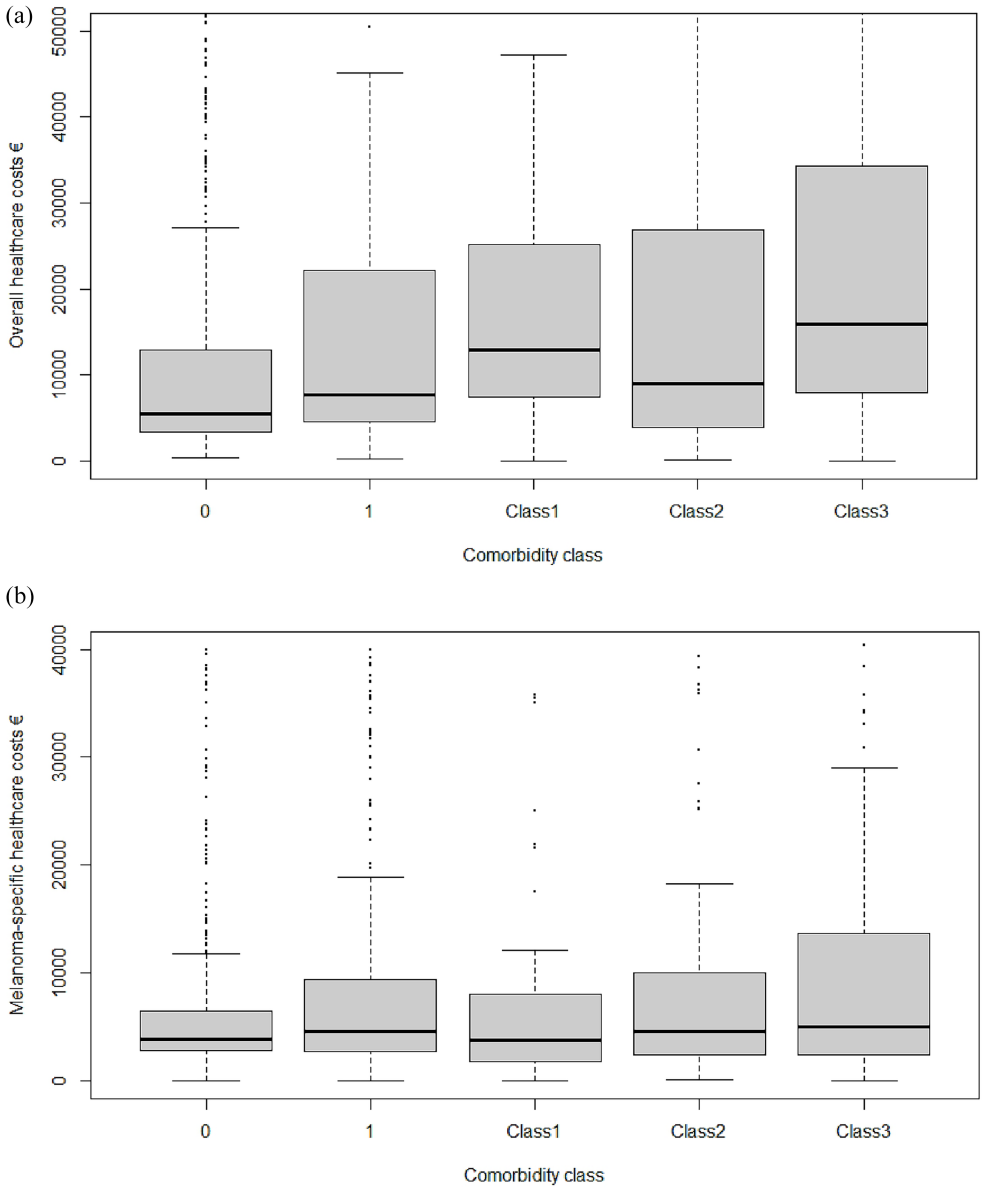
Overall **(a)** and melanoma-specific **(b)** cost differences across comorbidity categories.

Mean unadjusted overall cumulative expenditure per patient without comorbidity was €17,239 (95% CI 16,771–17,708) (Table 3, Figure 1a). Introducing a single chronic disease increased spending by 39% to € 24,014. Among multimorbidity classes, average costs were € 26,154 (Class 1), € 25,854 (Class 2), and € 33,222 (Class 3), the latter representing a 93% surcharge versus the reference cohort. A patient in the Multiorgan-Trauma class (Class 3) consumes €15,982 more than a comorbidity-free counterpart over 3 years. Hospitalizations were the dominant driver of cost escalation, rising from € 4,377 in patients without comorbidities to € 10,233 (Class 1) and € 11,355 (Class 3). Notably, medical-device expenditure increased 7-fold in Class 3.

**Table 3:** Three-year overall healthcare costs (all-cause) by comorbidity category (€, mean per patient).

Cost category (3-year, €)	No comorbidity	1 comorbidity	Class 1	Class 2	Class 3	Overall	*p*-value
			Circulatory-metabolic-respiratory	Psychosocial-Pregnancy related	Multiorgan-Trauma		
N	1,066	628	97	136	187	2,114	
Hospitalizations	4377.09	6609.76	10232.92	8381.15	11355.14	4899.83	<0.001^***^
Hospital drugs	7521.49	10666.23	8482.17	9432.46	9443.67	6848.68	<0.001^***^
Community drugs	563.10	653.60	1400.11	817.22	1256.82	643.68	<0.001^***^
Out-patient / specialist	3652.30	4611.43	4266.63	4379.13	3498.35	3391.73	<0.001^***^
Emergency room	75.86	95.65	175.45	129.94	193.52	89.68	0.006^**^
Hospice	42.39	57.96	205.64	16.86	290.11	59.87	<0.001^***^
Medical devices	1007.09	1319.83	1391.19	2697.60	7184.06	1668.58	<0.001^***^
Total	17239.32	24014.47	26154.12	25854.35	33221.67	17602.05	<0.001^***^

^*^p < 0.05; ^**^p < 0.01; ^***^p < 0.001.

The gradient was maintained when analyses were restricted to melanoma-specific health care resources: € 13,537 (no comorbidity) versus € 20,396 (Class 3). Systemic anticancer drugs consumed approximately 55% of melanoma-specific budgets in every group (*p* = 0.34) (Table 4, Figure 1b).

**Table 4:** Three-year melanoma-specific healthcare costs by comorbidity category (€, mean per patient).

Cost category (3-year, €)	No comorbidity	1 comorbidity	Class 1	Class 2	Class 3	Overall	*p*-value
			Circulatory-metabolic-respiratory	Psychosocial-pregnancy related	Multiorgan-trauma		
N	1,066	628	97	136	187	2,114	
Hospitalizations	2037.82	2336.36	1897.15	2455.78	1742.09	1583.65	<0.001^***^
Hospital drugs	6914.44	8814.30	6553.46	8343.68	8003.00	5825.94	0.342
Out-patient / specialist	3551.75	4313.45	3810.74	4248.03	3320.53	3239.45	<0.001^***^
Emergency room	1.08	3.27	2.62	5.35	7.05	2.23	0.043^*^
Hospice	24.99	40.97	123.37	16.86	139.35	37.71	0.116
Medical devices^a^	1007.09	1319.83	1391.19	2697.60	7184.06	1668.58	<0.001^***^
Total	13537.17	16828.17	13778.53	17767.30	20396.08	12357.56	0.034^*^

a For medical devices costs, no specific differentiation was made for melanoma in the original dataset, so overall values were used.

^*^p < 0.05; ^**^p < 0.01; ^***^p < 0.001.

In Tobit models left-censored at zero, Class 3 Multiorgan-Trauma and Class 2 Psychosocial-Pregnancy related multimorbidity significantly increase all-cause costs (Table 5). When the melanoma-specific cost was analyzed, Class 1 Circulatory-Metabolic-Respiratory multimorbidity (Table 6) was associated with a significant decrement (*p* = 0.013). Supplementary Tables S1, S2 present the Tobit regression analysis, which has been further adjusted to account for both medical and surgical treatments. Supplementary Table S1 indicates that patients with comorbidities incur higher overall costs, even after accounting for medical and surgical interventions. However, when examining melanoma-specific costs following this adjustment, no statistically significant differences were observed among different comorbidity classes.

**Table 5:** Tobit regression for overall healthcare costs (coefficients in €000) with hospital clustering.

Variable	Coef.^a^ (€000)	SE	95%CI	*p*-value
Intercept	5.76	2.24	(1.36, 10.16)	0.010^*^
One comorbidity	2.48	1.49	(−0.45, 5.41)	0.098
Class1	−0.26	2.96	(−6.06, 5.54)	0.930
Class2	5.69	3.00	(−0.19, 11.57)	0.058
Class3	6.94	2.57	(1.89, 11.98)	0.007^**^
Male sex	3.12	1.40	(0.37, 5.87)	0.026^*^
Age 45–59	−0.15	2.16	(−4.39, 4.09)	0.945
Age 60–74	3.29	2.68	(−1.97, 8.54)	0.220
Age ≥75	3.69	2.29	(−0.79, 8.17)	0.106
Stage II	11.67	1.80	(8.15, 15.19)	<0.001^***^
Stage III	48.25	2.70	(42.95, 53.54)	<0.001^***^
Stage IV	24.72	4.88	(15.16, 34.28)	<0.001^***^

Reference: comorbidity: 0 comorbidity (only tumor). Sex: female. Age: <45. Stage: I.

^a^Coefficients are expressed in thousands of euros (€000). ^*^p < 0.05; ^**^p < 0.01; ^***^p < 0.001.

**Table 6:** Tobit regression for melanoma-specific healthcare costs (coefficients in €000) with hospital clustering.

Variable	Coef.^a^ (€000)	SE	95%CI	*p*-value
Intercept	4.38	1.52	(1.41, 7.36)	0.004^**^
One comorbidity	−0.40	1.22	(−2.79, 1.99)	0.744
Class 1	−6.48	2.60	(−11.58, −1.38)	0.013^*^
Class 2	1.15	2.79	(−4.31, 6.62)	0.680
Class 3	0.59	2.51	(−4.33, 5.51)	0.814
Male sex	2.76	0.99	(0.81, 4.70)	0.005^**^
Age 45–59	−0.10	1.51	(−3.06, 2.85)	0.945
Age 60–74	0.95	2.20	(−3.36, 5.25)	0.666
Age ≥75	−0.69	1.88	(−4.37, 2.99)	0.714
Stage II	11.16	1.49	(8.24, 14.08)	<0.001^***^
Stage III	45.26	2.51	(40.34, 50.17)	<0.001^***^
Stage IV	23.02	4.56	(14.09, 31.95)	<0.001^***^
Stage missing	12.24	2.95	(6.46, 18.03)	<0.001^***^

Reference: comorbidity: 0 comorbidity (only tumor). Sex: female. Age: <45. Stage: I.

^a^Coefficients are expressed in thousands of euros (€000). ^*^p < 0.05; ^**^p < 0.01; ^***^p < 0.001.

## 4 Discussion

Our findings suggest that melanoma costs increase sharply with multimorbidity complexity, and each comorbidity pattern is associated with a distinct economic burden.

A growing body of evidence indicates that the cost of cancer care is influenced not only by tumor stage and therapeutic choices, but also—and often decisively—by the bundle of chronic illnesses a patient has (21). However, there is still a lack of evidence regarding how co-existing comorbidities affect the economics of melanoma care. Among the few available studies is the work of Birch et al. (22), which analyzed the additional costs associated with anxiety and depression in Medicare beneficiaries with melanoma and other cancers. A series of cohort studies shows a consistent pattern: whenever advanced stage coincides with several comorbidities, costs rise sharply, and even common disorders such as diabetes or hypertension add a substantial surcharge to hospital costs (23, 24).

Our results provide robust evidence that multimorbidity clusters are associated with distinct healthcare cost trajectories in melanoma. Multiorgan-Trauma and Psychosocial-Pregnancy related multimorbidity inflate overall healthcare costs whereas Circulatory-Metabolic-Respiratory multimorbidity is associated with lower melanoma-specific spending in adjusted models. The lower melanoma-specific clinical pathway costs in the Circulatory-Metabolic-Respiratory cluster, as revealed in the multivariate analysis, reflect the de-intensification of medical therapy in patients with competing health risks that we observed in the univariate analysis (Table 1), particularly among patients with Class 1 comorbidities. One possible explanation is that poor performance status or patients receiving concurrent chronic steroid therapy may contraindicate immunotherapy (25–27). Nevertheless, although there is robust evidence indicating that the administration of cytotoxic chemotherapy in patients with poor performance status is linked to increased toxicity—thereby overshadowing potential efficacy—the effects of performance status on the safety and efficacy of immunotherapy remain unclear. Since the side effect profile of checkpoint inhibitors differs markedly from that of chemotherapy, immunotherapy may represent a preferable option for patients for whom the only alternative would be best supportive care (28). Contraindications to targeted cancer therapy could be varied and include pre-existing heart conditions, uncontrolled high blood pressure, or uncontrolled asthma, which could affect patients in this comorbidity group more. The development and approval of multidisciplinary clinical practice guidelines tailored to different comorbidities should be a focus of future efforts. This will help clinicians apply the best evidence based on patients’ clinical conditions especially for the most common comorbidities, like heart failure, diabetes, and chronic obstructive pulmonary disease.

Our study suggests that incorporating model-level information into hospital budgeting and cost-effectiveness models can improve the accuracy of resource forecasting and value-based care assessment.

Further research could also evaluate how recognition of comorbidity patterns could help provide precise, supportive care for optimal management of these patients and reduce the healthcare burden, for example, perioperative cardiopulmonary optimization for the cardiorespiratory class, coordinated maternity and psychosocial management for the psychosocial-pregnancy-related class, and targeted vaccination plus antimicrobial prophylaxis for the Multiorgan-Trauma class.

The strengths of this study encompass its real-world design which incorporates a large, population-based, high-quality registry linked with administrative data. Additionally, the study introduces the innovative application of Latent Class Analysis (LCA) to identify comorbidity clusters among melanoma patients.

The study also acknowledges certain limitations. Our retrospective investigation is confined to a single region in Northeast Italy where healthcare services are provided under a publicly funded system; therefore, the generalizability of the results is limited, and prudence should be exercised when applying these findings to different healthcare contexts. To extend and validate our findings more broadly, multi-country prospective studies would be necessary.

The present results focused exclusively on direct healthcare costs accounted for by the national health system. Indirect costs, such as productivity loss, informal caregiving, and societal burden, were not included in the analysis. Incorporating these factors in future analyses could provide a more comprehensive understanding of the total cost burden according to comorbidity class.

The presence of comorbidities was determined from hospital discharge records not using algorithms based on other administrative databases such as medication records or exemptions. This approach might miss conditions that are not severe enough to be recorded during hospitalization, leading to potential underestimation which could bias the cohort toward sicker patients. Consequently, the actual comorbidity burden could be underestimated in its prevalence, although its impact on healthcare costs may be overestimated. Incorporating algorithms that identify chronic diseases from additional administrative records, beyond hospital discharge data, could improve the detection of mild conditions, and help refine cluster definitions.

Furthermore, there was a selection bias within the cohort, as only patients with hospital records were included. This group does not accurately represent the entire melanoma patient population, particularly as it excludes patients who underwent wide local excision in an outpatient setting—typically representing lower-stage and younger patients (data not shown). Such bias may lead to an overestimation of the comorbidity burden within the melanoma patient cohort and its associated impact on healthcare costs.

The choice to analyze the first hospitalization occurring 6 months after diagnosis was made to ensure a timeframe that includes the most likely period when the wide excision surgery was scheduled following the biopsy diagnosis, which is instead an outpatient procedure. Comorbid conditions diagnosed within a few months of a cancer diagnosis might be part of the pre-diagnostic conditions; however, in some cases, they may capture treatment adverse events rather than actual baseline comorbidities. We cannot rule out the possibility of misclassifying comorbid conditions, which could lead to inflated costs attributed to comorbidity.

## Data Availability

The data supporting this study’s findings are held by the Veneto Tumour Registry and were used under license for this work. The anonymized minimal data set necessary to replicate our findings has been made publicly available at the following link: https://doi.org/10.6084/m9.figshare.29381702.
