# Supplementary material for: Healthcare costs of cutaneous melanoma according to comorbidity patterns: a population-based study from the Regional Cancer Registry of the Veneto Region
Source: Front Public Health. 2025 Oct 17;13:1668198. doi: 10.3389/fpubh.2025.1668198 (PMC12576802; doi:10.3389/fpubh.2025.1668198)
Supplement: Supplementary file 1 [file Table_1.docx]

Supplementary Material

# Supplementary Tables

## Supplementary Table S1. Tobit regression (adjusted for sex, age, stage at diagnosis, and surgical medical treatment variables) for overall healthcare costs (coefficients in €000).

| **Variable** | **Coef. (€000)** | **SE** | **95% CI** | **p-value** |
| --- | --- | --- | --- | --- |
| (Intercept) | 2.611 | 2.544 | (-2.376, 7.597) | 0.305 |
| Comorbidity 1 | 3.149 | 1.263 | (0.673, 5.625) | 0.013^a^ |
| Comorbidity Class 1 | 1.289 | 2.718 | (-4.039, 6.617) | 0.635 |
| Comorbidity Class 2 | 5.735 | 2.289 | (1.249, 10.221) | 0.012 ^a^ |
| Comorbidity Class 3 | 8.238 | 2.074 | (4.174, 12.302) | <0.001 ^c^ |
| Sex Male | 2.160 | 1.134 | (-0.062, 4.383) | 0.057 |
| Age 45-59 | -0.372 | 1.889 | (-4.073, 3.330) | 0.844 |
| Age 60-74 | 4.059 | 1.864 | (0.406, 7.712) | 0.029* |
| Age 75+ | 7.948 | 2.004 | (4.020, 11.876) | <0.001 ^c^ |
| TNM Stage II | 8.740 | 1.611 | (5.582, 11.899) | <0.001 ^c^ |
| TNM Stage III | 24.090 | 2.511 | (19.168, 29.012) | <0.001 ^c^ |
| TNM Stage IV | 5.895 | 3.082 | (-0.145, 11.935) | 0.056 |
| TNM Stage Missing | 1.725 | 3.141 | (-4.431, 7.881) | 0.583 |
| Medical Therapy Only Immunotherapy | 34.960 | 3.303 | (28.489, 41.435) | <0.001 ^c^ |
| Medical Therapy Only Target therapy | 48.910 | 3.377 | (42.295, 55.533) | <0.001 ^c^ |
| Medical Therapy Target & Immunotherapy | 49.500 | 14.630 | (20.830, 78.173) | <0.001 ^c^ |
| Lymphadenectomy | 1.075 | 1.231 | (-1.338, 3.489) | 0.382 |
| Wide Expansion | 1.164 | 1.886 | (-2.533, 4.861) | 0.537 |
| BRAF Mutation Mutated | 10.960 | 2.646 | (5.771, 16.142) | <0.001 ^c^ |
| BRAF Mutation Not mutated | 4.501 | 2.331 | (-0.067, 9.068) | 0.053 |
| BRAF Mutation Not recovered | 1.698 | 3.797 | (-5.743, 9.139) | 0.655 |
| BRAF Mutation Missing | 4.953 | 3.067 | (-1.059, 10.964) | 0.106 |

**Note:** Coefficients are expressed in thousands of euros (€000). Significance levels: ^a^ p<0.05, ^b^ p<0.01, ^c^ p<0.001

Reference: Comorbidity: 0 Comorbidity (only tumor). Sex: Female. Age:<45. Stage: I. Therapy: None. BRAF Mutation: Not performed

## Supplementary Table S2. Tobit regression (adjusted for sex, age, stage at diagnosis, and surgical medical treatment variables) for melanoma‑specific healthcare costs (coefficients in €000).

| **Variable** | **Coef. (€000)** | **SE** | **95% CI** | **p-value** |
| --- | --- | --- | --- | --- |
| (Intercept) | 0.284 | 2.130 | (-3.891, 4.459) | 0.894 |
| Comorbidity 1 | 0.372 | 1.057 | (-1.701, 2.444) | 0.725 |
| Comorbidity Class 1 | -4.300 | 2.278 | (-8.765, 0.164) | 0.059 |
| Comorbidity Class 2 | 1.581 | 1.915 | (-2.172, 5.335) | 0.409 |
| Comorbidity Class 3 | 2.338 | 1.737 | (-1.065, 5.742) | 0.178 |
| Sex Male | 1.573 | 0.949 | (-0.287, 3.433) | 0.097 |
| Age 45-59 | -0.403 | 1.581 | (-3.502, 2.695) | 0.799 |
| Age 60-74 | 1.515 | 1.560 | (-1.543, 4.573) | 0.332 |
| Age 75+ | 3.435 | 1.678 | (0.145, 6.724) | 0.041 ^a^ |
| TNM Stage II | 8.486 | 1.348 | (5.843, 11.128) | <0.001 ^c^ |
| TNM Stage III | 21.950 | 2.102 | (17.826, 26.066) | <0.001 ^c^ |
| TNM Stage IV | 5.453 | 2.586 | (0.385, 10.521) | 0.035 ^a^ |
| TNM Stage Missing | 5.655 | 2.633 | (0.494, 10.815) | 0.032 ^a^ |
| Medical Therapy Only Immunotherapy | 33.720 | 2.764 | (28.302, 39.138) | <0.001 ^c^ |
| Medical Therapy Only Target therapy | 49.130 | 2.826 | (43.592, 54.671) | <0.001 ^c^ |
| Medical Therapy Target & Immunotherapy | 44.150 | 12.240 | (20.154, 68.137) | <0.001 ^c^ |
| Lymphadenectomy | 0.793 | 1.030 | (-1.227, 2.812) | 0.442 |
| Wide Expansion | 2.557 | 1.579 | (-0.538, 5.652) | 0.105 |
| BRAF Mutation Mutated | 9.346 | 2.214 | (5.006, 13.687) | <0.001 ^c^ |
| BRAF Mutation Not mutated | 5.768 | 1.951 | (1.945, 9.591) | 0.003 ^b^ |
| BRAF Mutation Not recovered | 1.095 | 3.184 | (-5.146, 7.336) | 0.731 |
| BRAF Mutation Missing | 3.985 | 2.567 | (-1.047, 9.017) | 0.121 |

**Note:** Coefficients are expressed in thousands of euros (€000). Significance levels: ^a^ p<0.05, ^b^ p<0.01, ^c^ p<0.001

Reference: Comorbidity: 0 Comorbidity (only tumor). Sex: Female. Age:<45. Stage: I. Therapy: None. BRAF Mutation: Not performed
